# Effects of two commercial diets and two supplements on urinary pH in dogs

**DOI:** 10.1002/vms3.1285

**Published:** 2023-10-18

**Authors:** Inês Alcaide Igreja, Ana Luísa Lourenço, Johannes C. M. Vernooij, Ronald Jan Corbee

**Affiliations:** ^1^ Department of Animal Science Universidade de Trás‐os‐Montes e Alto Douro (UTAD) Vila Real Portugal; ^2^ Department of Animal Science, Quinta de Prados Animal and Veterinary Research Center (CECAV) Vila Real Portugal; ^3^ Department of Population Health Sciences, Faculty of Veterinary Medicine Utrecht University Utrecht The Netherlands; ^4^ Department of Clinical Sciences, Faculty of Veterinary Medicine Utrecht University Utrecht The Netherlands

**Keywords:** Bladder, canine, food, nutrition, urine, urolithiasis

## Abstract

**Background:**

Urinary pH manipulation by therapeutic foods or supplements is part of the treatment for urolithiasis. The effectiveness of these diets and supplements should be studied to determine which of these strategies is most effective.

**Hypothesis/Objectives:**

To assess the effect of the oral supplementation of potassium citrate, an ammonium chloride solution (Urical) and two dry therapeutic foods—Hill's® Prescription Diet® u/d® Canine (u/d diet) and Royal Canin® Urinary S/O dog (S/O diet)—on a dog's urinary pH at different time points over 8 h.

**Animals:**

Seven healthy adult male research beagle dogs.

**Methods:**

A prospective interventional study lasting 31 days. The dogs either received a supplement (potassium citrate or rical) with a dry adult maintenance diet (control diet) or the therapeutic diet (u/d diet or S/O diet). Each treatment had a duration of 2–5 days, with 2‐ to 4‐day washout periods in between. Urinary pH measurements were performed every 2 h between 07h00 and 15h00, with the food being given at 07h00 and 15h00, right after urine collection. The pH measurements obtained in each of the four treatments were compared to control (same dogs fed the control diet exclusively).

**Results:**

When compared to the control diet at the same time points, biologically relevant changes in urinary pH (defined as ≥0.5) were: increase with potassium citrate at 7h00 and 13h00; increase with u/d diet at 9h00, 13h00, and 15h00; decrease with S/O diet at 9h00 and 11h00; Urical did not have a detectable effect on urinary pH.

**Conclusions and Clinical Importance:**

The present study confirms that therapeutic foods S/O and u/d, and potassium citrate supplement affected acid‐base balance in healthy adult male beagle dogs, with the tested diets being more effective than the administered doses of the tested supplements at influencing urinary pH.

## Graphical abstract

The present study confirms that therapeutic foods S/O and u/d and potassium citrate supplement (65–105 mg/kg BW/2dd) affected acid‐base balance in healthy adult male Beagle dogs, by a change of urinary pH ≥0.5 at different time points during the alkaline tide after a meal. Urical (ammonium chloride 9 mg/kg per day) did not have a detectable effect on urinary pH. The tested diets were more effective than the administered doses of the tested supplements at influencing urinary pH.

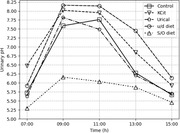

## 1 INTRODUCTION

Urinary pH can be regarded as an estimation of the acid‐base balance (Barsanti, 2012). Urinary pH is not constant and fluctuates during the day. There is an indication of a physiologic acid‐base rhythm with a curve of diurnal fluctuation in both humans, cats and dogs (Allen, 1996; Elliot et al., 1959; Stevenson & Markwell, 2001), which is characteristic for a given individual, and is a result of several factors such as diet and the number of meals per day, emotional status, exercise, personality and pulmonary ventilation (Allen, 1996; Elliot et al., 1959). The alkalization of the blood and urine that results from the physiological process that follows a meal is designated the alkaline tide effect (Niv & Fraser, 2002). Food and endogenous metabolic processes are the sources of acid or base intake and production, and so it is possible to efficiently alter or adjust the urinary pH by dietetic means only (Dwyer et al., 1985; Remer & Manz, 1994). Urinary pH manipulation is beneficial in multiple conditions but is particularly important in preventing the formation of some types of uroliths (Lulich et al., 2016). Two main strategies are used to influence urinary pH: oral supplements that produce alkaline and acidic urine, such as potassium citrate and ammonium chloride, respectively, and alkalizing or acidifying therapeutic foods. Numerous therapeutic foods are formulated to prevent the recurrence of uroliths in dogs. The potential of a diet to acidify or alkalize the urine depends on its ingredients and the equilibrium between acidifiers, such as methionine, calcium sulfate, ammonium chloride and alkalizers, such as calcium carbonate and potassium citrate (Queau, 2019).

Some studies evaluating the effects of oral supplements added into regular canine diets, such as potassium citrate (Stevenson et al., 2000), dl‐methionine (Girardi et al., 1990), calcium sulfate (Janczikowski et al., 2008), or ammonium chloride (Senior et al., 1984; Shaw, 1989) are available. The effects of therapeutic foods formulated to prevent the recurrence of uroliths (Lulich et al., 2016) were also performed. Nevertheless, studies evaluating the influence of supplements and diets on urinary pH in dogs are scarce.

The aim of the present study was to: assess the effect of day and time on the urinary pH profile; and assess the effect of potassium citrate, a solution containing ammonium chloride (Urical), and two therapeutic foods: Hill's® Prescription Diet® u/d® Canine (u/d diet, target pH: 7.1–7.7) and Royal Canin® Urinary S/O dog (S/O diet, target pH: 6.0–6.5) on the dog's urinary pH, including their effect on the alkaline tide, which according to the authors’ knowledge has not been studied before. U/d diet is formulated to reduce the risk of oxalate, urate and cystine stone formation and S/O diet is formulated to reduce the risk of struvite and oxalate stone formation. The hypotheses were that (1) the u/d diet and potassium citrate (130–211 mg/kg BW/day divided over 2 doses per day) would produce a higher urine pH versus control (dogs fed solely a dry adult maintenance diet) and (2) that the S/O diet and Urical (0.5 mL/kg BW/day) would produce a lower urine pH versus control.

## 2 MATERIALS AND METHODS

### 2.1 Ethics approval

The study protocol was reviewed and approved by the Clinical Research Review Committee at Utrecht University, registered under number AVD1080020184748, as required by Dutch legislation.

### 2.2 Dogs

Seven research Beagle dogs were selected to take part in the study. All dogs were male and intact, with ages between 1 and 5 years old, weighing 11.3 ± 2.15 kg (±SD) and having a BCS of 5/9. Based on a physical examination, a complete blood count, serum biochemistry and a complete urinalysis, the dogs were determined to be healthy. The dogs were housed in their normal kennel environment within the same pairs and groups to minimize stress. The kennels had an indoor surface of 172 × 240 × 225 (length × width × height in centimetres) and an outdoor surface of 242 × 315 × 178, to both of which the dogs had unlimited access. The indoor temperature was kept constant at 20°C and the relative humidity at 65% by the kennel climate system. Five times per week, the dogs were left for 2.5 h in playpens with a surface area of 400 × 500 × 198. The dogs were housed in pairs, and some were housed individually for behavioural reasons.

### 2.3 Study design

**FIGURE 1:**
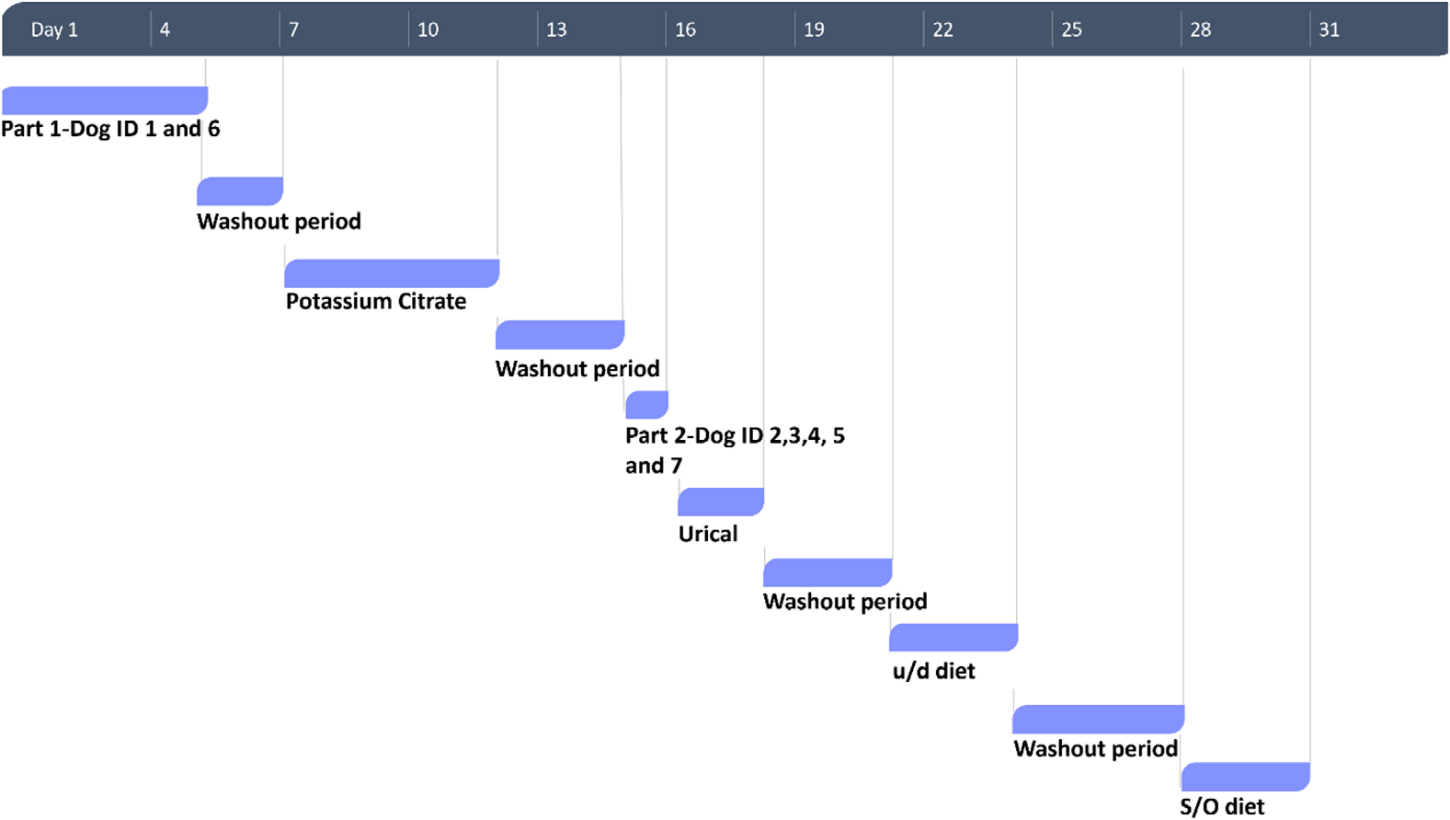
Study design timeline. Part 1–control diet (dog ID 1 and 6); Part 2–control diet (dog ID 2,3,4,5, and 7); Potassium citrate—potassium citrate supplement; Urical—ammonium chloride solution; u/d diet—Hill's ® Prescription Diet® u/d® Canine; S/O diet—Royal Canin® Urinary S/O dog. During the washout periods the dogs were fed the control diet.

The study timeline can be consulted in Figure 1. The study was conducted over 31 days and set up in three parts. In Part 1, as a pilot phase, urinary pH was measured in two dogs (dog ID 1 and 6) at 2‐h intervals between 7h00 and 15h00 (time 0, 2, 4, 6, 8) for five consecutive days, when fed a dry maintenance diet for adult dogs, Hill's® Science Plan® Adult Medium Breed Advanced Fitness Lamb and Rice (control diet, Tables 1 and 2). An additional measurement at 17h00 on the first day and 10h00 on the last 4 days were collected to decide the best time points for urine sample collection throughout the experiment, but they were removed from the results for study design clarity. This pilot was to determine or confirm the optimal measurement intervals and to determine if there were changes in pH between consecutive days.

**TABLE 1:** Reported nutrient composition by manufacturers of one maintenance adult dog diet (control diet) and two therapeutic foods formulated to prevent recurrence of calculi in dogs.

Variable	Hill's science plan (control diet)^a^	U/d diet^b^	S/O diet^c^
Protein (g/100 kcal)	5.7	2.5	4.7
Fat content (g/100 kcal)	3.8	4.8	4.4
Carbohydrate (g/100 kcal)	13.4	14.4	11.2
Crude fiber (g/100 kcal)	0.4	0.6	0.59
Crude ash (g/100 kcal)	1.3	0.8	1.7
Sodium (g/100 kcal)	0.064	0.054	0.31
Calcium (g/100 kcal)	0.205	0.104	0.13
Potassium (g/100 kcal)	5.7	0.143	0.21
Phosphorus (g/100 kcal)	3.8	0.042	0.13
Magnesium (g/100 kcal)	13.4	0.013	0.013
Moisture (%)	8	7.5	9.5
Metabolisable energy (kcal/kg)	3718	3998	3866

^a^ Hill's science plan adult medium breed advanced fitness lamb and rice, Hill's pet nutrition.

^b^ Hill's prescription diet u/d canine, Hill's pet nutrition.

^c^ Royal Canin canine urinary S/O, Royal Canin.

**TABLE 2:** Ingredients of one maintenance adult dog diet (control diet), two therapeutic foods formulated to prevent recurrence of calculi in dogs, and one ammonium chloride containing solution.

Diet/Supplement	Ingredients	Dosage
Hill's science plan (control diet)^a^	Maize, wheat, lamb meal, soybean meal, animal fat, maize gluten meal, brewers' rice, protein hydrolysate, soybean oil, linseed oil, minerals, beta‐carotene.	
U/d diet^b^	Brewers rice, corn starch, animal fat, egg powder, cellulose, minerals protein hydrolysate, linseed, dried beetpulp, soybean oil, trace elements, L‐carnitine, vitamins, taurine, beta‐carotene.	
S/O diet^c^	Corn meal, rice, animal fat, dehydrated poultry proteins, maize gluten, minerals, hydrolysate of animal proteins, plant fibre, soybean oil, fish oil, fructo‐oligo‐saccharides, mono‐ and diglycerides of palmitic acid and stearic acid esterified with citric acid, tagetes extract.	
Urical	Ammonium chloride, citric acid, iron chloride, glucose, potassium chloride, methionine, methylthionine, sodium chloride, flavouring agents, water, zinc sulphate.	0.5 mL/kg BW/day
Potassium citrate	Potassium citrate	130–211 mg/kg BW/day divided over 2 doses per day

^a^ Hill's science plan adult medium breed advanced fitness lamb and rice, Hill's pet nutrition.

^b^ Hill's prescription diet u/d canine, Hill's pet nutrition.

^c^ Royal Canin® canine urinary S/O, Royal Canin.

In Part 2, urinary pH was measured in five other dogs (dog ID 2,3,4,5 and 7) fed the control diet for 1 day at 2‐h intervals between 7h00 and 15h00 (time 0, 2, 4, 6, 8). The data from this part and from the first 2 days of Part 1 (dogs ID 1 and 6) were used to establish the control urinary pH (Control) used to compare the results of every treatment.

In Part 3, all seven dogs either received a supplement (potassium citrate or Urical) with the control diet or the therapeutic diet (u/d diet or S/O diet) fed over two equal meals at 07h00 and 15h00, right after urine collection via natural voiding. There was a minimum washout period of 2 days between every treatment, enough to eliminate residual effects of the previous treatment, and a maximum of 4 days needed due to logistic restrictions. All dogs followed the same order of treatments and washout periods for logistical reasons. During the washout periods, the dogs were fed the control diet. The amount of food was calculated according to the adult maintenance energy requirements for neutered dogs MER = 1.6 × (body weight in kg)^0.75^ × 70 in kcal ME per day (Thatcher et al., 2010), and water was provided ad libitum. Dogs readily consumed all the food provided. Urine samples were collected every two hours between 07h00 and 15h00. All urinalysis results were obtained from urine samples collected spontaneously during a walk. The samples were collected using a cardboard cup, after the first drops of urine were voided.

Potassium citrate was administered for 5 days at 130–211 mg/kg BW/day divided over two doses per day. The doses were chosen based on the literature (Adams & Syme, 2010; Stevenson et al., 2000). A capsule form was chosen (Fagron—Kaliumcitraat), with each capsule containing 500 mg of potassium citrate. During the first three days (KCit1), the potassium citrate was supplemented at 07h00 and 15h00 with the meal. To further study the potassium citrate supplementation effect apart from the meal, because there is already an alkaline tide following a meal, supplementation during that alkaline tide can prolong the alkaline tide. During the last 2 days of this treatment, the potassium citrate was given 3.5 h after the first meal (KCit2); thus, supplementation took place at 10h30 and 17h30. Uricals were administered for 2 days. According to the manufacturer's instructions, the dosage of Urical was 0.5 mL/kg BW/day. Because the manufacturer was not willing to provide the dosage of ammonium chloride in this product, we analysed the amount of nitrogen by the Dumas method, resulting in a level of 13.43 g per kg of product. The maximum dosage of ammonium chloride in Urical is therefore 0.009 g/kg BW/day (nitrogen has a molar weight of 14 g and ammonium chloride has a molar weight of 18 g, so 13.43 g nitrogen is present in 17.3 g of ammonium chloride. This is in 1 kg, which is approximately 1000 mL, so in 0.5 mL there will be about 0.009 g of ammonium chloride). Urine samples were collected on both days. U/d was administered for 3 days. According to the manufacturer, this diet is formulated to induce alkaline urine (target pH: 7.1–7.7). Urine samples were collected during the last 2 days. S/O diet was administered for 3 days. According to the manufacturer, this diet is formulated to induce acidic urine (target pH: 6.0–6.5). Urine samples were collected during the last 2 days.

### 2.4 Urinalysis

The urine samples were stored in an Eppendorf tube, briefly, at room temperature (approx. 18°C) until analysis, which was performed within 3 h after sample collection. The urine samples were first centrifuged to remove the cell pellet, and then the supernatant was analysed for pH using a bench‐top pH meter (HI 2209 pH meter, Hanna Instruments).

### 2.5 Statistical analysis

The urinary pH of dogs 1 and 6 (Part 1) was analysed with a general linear model with the factors day, time, the interaction between day and time and dog. Visual inspection of residuals was used to check the model assumptions and did not show any abnormalities. The Akaike's information criterion (AIC) was used to test the importance of the interaction, resulting in its removal from the model due to its non‐significant effect. Although day could be removed from the model, it was kept in the model to show the difference between days, as this is an important question to answer to proceed with Part 2. Model results are presented as estimates for the difference between means with 95% confidence intervals. Outcome variable urinary pH values in KCit1, Urical, u/d diet and S/O diet, were analysed by a mixed effects linear regression model (Pinheiro et al., 2021) with factors Treatment, Time and the interaction between both. For the analysis only measurements of Days 1 and 2 at Time 0, 2, 4, 6 and 8 of each treatment were used. The data from the first 2 days of Part 1 and from Part 2 were used to establish the control urinary pH (Control) used to compare the results of every treatment. Control and Time 0 were used as references for comparisons. To account for the correlation between repeated measurements, dog ID was added as a random effect, and a random slope per dog was added to the model for the correlation between time points. The AIC was used to select the best model. Statistical package R version 4.0.5 (R Core Team, 2021) was used for calculations. Results were presented as differences between means with 95% confidence intervals. Model assumptions were studied by a visual check of residual plots, which did not show any abnormalities.

The comparison of urinary pH values in KCit2 was similarly analysed as the model for comparing the other treatments. The difference was that less sampling was done for KCit2 (11, 13 and 15 h). To account for the correlation between repeated measurements dog ID was added as random effect and an Auto Regressive correlation (AR1) was added to the model for the correlation between time points. The same modelling procedure was applied as previously described model. Results were presented as differences between means with 95% confidence intervals. Model assumptions were studied by the visual check of residual plots, which did not show any abnormalities. A difference of at least 0.5 in urinary pH was considered a biologically relevant difference. Graphic representations were created using a plotting library called Matplotlib for the Python 3.6 programming language and its numerical mathematics extension, NumPy.

## 3 RESULTS

Estimated differences between means and 95% confidence intervals of specific day and time of the urinary pH measured in dogs 1 and 6 over 5 days is represented in Table 3. There was no significant difference in urinary pH at the different time points between days.

**TABLE 3:** Estimated differences between means and 95% confidence intervals of specific Day and Time of the urinary pH measured in 2 dogs when fed the control diet over 5 days (Part1).

		95% Confidence interval	
	Estimate^b^	2.5%	97.5%
Intercept^a^	5.63	5.25	6.01
Day 2 versus Day 1	−0.05	−0.43	0.34
Day 3 versus Day 1	0.14	−0.25	0.52
Day 4 versus Day 1	0.19	−0.19	0.58
Day 5 versus Day 1	0.29	−0.09	0.68
T2 versus T0	1.71	1.32	2.09^*^
T4 versus T0	1.89	1.5	2.27^*^
T6 versus T0	0.44	0.06	0.83^*^
T8 versus T0	−0.19	−0.57	0.2
Dog 6 versus Dog 1	−0.14	−0.39	0.1

^a^ Estimated mean in the reference: Day 1 and Time 0 (7h00) and dog 1. T0 is the time of feeding a meal (7h00), T2 is 2 h after the meal (9h00), T4 is 4 h after the meal (11h00), T6 is 6 h after the meal (13h00) and T8 is 8 h after the meal (15h00).

^b^ Estimated difference between mean of specific Day, Time and Dog respectively with reference.

* Significant and biologically relevant (i.e., pH change of at least 0.5).

**FIGURE 2:**
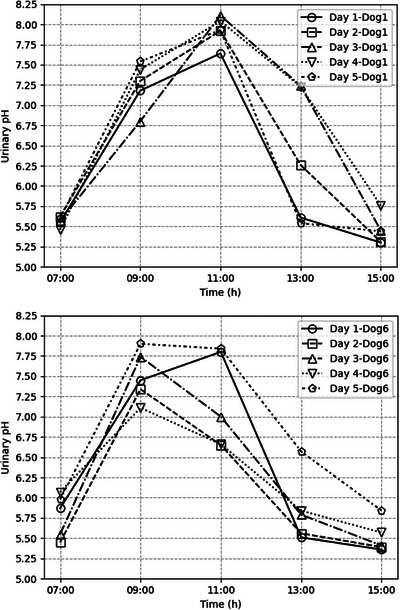
Urinary pH values for dogs 1 and 6 respectively, when fed the control diet for 5 consecutive days, divided over two meals per day, given at 07h00 and 15h00 (Part 1).

Urinary pH values for dogs 1 and 6, when fed the control diet for five consecutive days (Part 1), are represented in Figure 2. An alkaline tide was present at 2, 4 and 6 h after meal (9h00, 11h00, 13h00), whereas urinary pH returned to baseline 8 h after meal (15h00).

The mean urinary pH [95% confidence interval] between 7h00 and 15h00 for control and the four treatments is represented in Table 4.

**TABLE 4:** Estimated differences between means and 95% confidence intervals of specific Time of the urinary pH measured in 7 dogs between control and each treatment: (Potassium Citrate administer at 07h00 (KCit1); Urical; u/d diet; S/O diet.

		95% Confidence interval	
	Estimate^b^	2.5%	97.5%
Intercept^a^	5.74	5.43	6.04
Control T2 versus T0	1.86	1.45	2.27^*^
Control T4 versus T0	2.03	1.62	2.45^*^
Control T6 versus T0	0.55	0.12	0.98^*^
Control T8 versus T0	−0.06	−0.51	0.39
KCit 1 versus Control at T0	0.75^2^	0.38	1.13^*^
KCit 1 versus Control at T2	0.43	0.07	0.8^#^
KCit 1 versus Control at T4	0.19	−0.18	0.55
KCit 1 versus Control at T6	0.57	0.2	0.94^*^
KCit 1 versus Control at T8	0.26	−0.11	0.63
Urical versus Control at T0	−0.1	−0.47	0.27
Urical versus Control at T2	0.23	−0.14	0.59
Urical versus Control at T4	−0.27	−0.64	0.1
Urical versus Control at T6	−0.07^2^	−0.44	0.3
Urical versus Control at T8	0.03	−0.34	0.4
u/d diet versus Control at T0	0.19	−0.18	0.56
u/d diet versus Control at T2	0.57	0.2	0.94^*^
u/d diet versus Control at T4	0.38	0.01	0.75^#^
u/d diet versus Control at T6	1.17	0.8	1.54^*^
u/d diet versus Control at T8	0.47	0.1	0.83^*^
S/O diet versus Control at T0	−0.43	−0.8	−0.06^#^
S/O diet versus Control at T2	−1.43	−1.8	−1.07^*^
S/O diet versus Control at T4	−1.72	−2.08	−1.35^*^
S/O diet versus Control at T6	−0.4	−0.77	−0.03^#^
S/O diet versus Control at T8	−0.21	−0.58	0.16

^a^ Estimated mean in the reference: Control and Time 0 (7h00). T0 is the time of feeding a meal (7h00), T2 is 2 h after the meal (9h00), T4 is 4 h after the meal (11h00), T6 is 6 h after the meal (13h00), and T8 is 8 h after the meal (15h00).

^b^ Estimated difference between mean of specific Time with reference.

* Significant and biologically relevant (i.e., pH change of at least 0.5).

^#^ Significant but not biologically relevant.

KCit1–potassium citrate administered at 7h00 and at 15h00; Urical—ammonium chloride solution; u/d diet—Hill's® Prescription Diet® u/d® Canine; S/O diet—Royal Canin® Urinary S/O dog.

**FIGURE 3:**
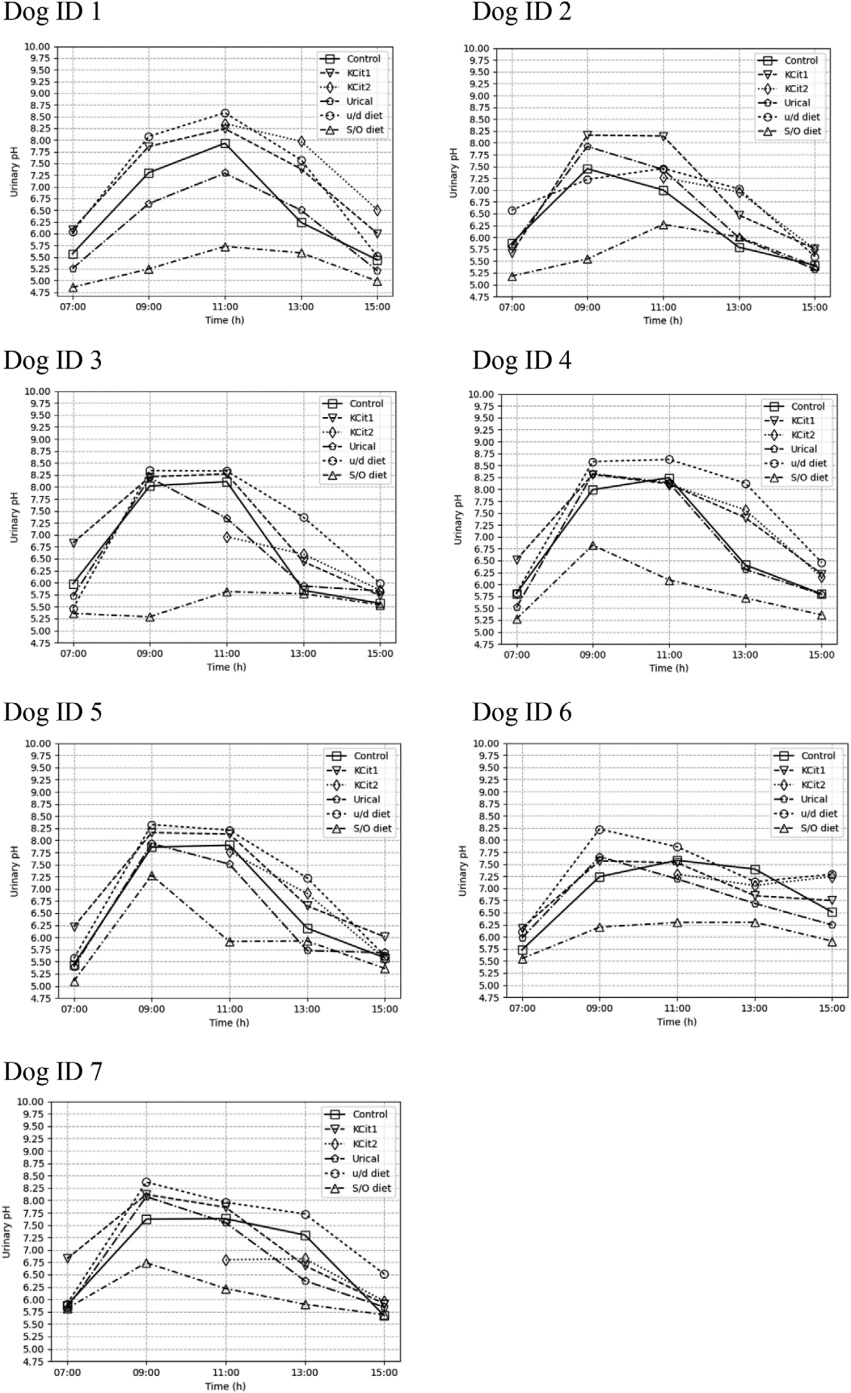
Mean urinary pH, for every dog individually, for control and per treatment: KCit1—potassium citrate administered at 07h00 and at 15h00; KCit2 – potassium citrate administered at 10h30 and at 15h00; Urical—ammonium chloride solution; u/d diet—Hill's ® Prescription Diet® u/d® Canine; S/O diet—Royal Canin® Urinary S/O dog.

Mean urinary pH, for every dog individually, for control and per treatment, are represented in Figure 3. An overall increase in mean urinary pH between 9h00 and 13h00 (approximately 2–6 h after meal) is also evident in most dogs, whereas urinary pH returned to baseline 8 h after meal (15h00). It can also be observed that u/d diet produced a higher mean urinary pH versus control and versus potassium citrate on almost every dog; and that S/O diet produced a lower mean urinary pH versus control and versus Urical, almost flattening the alkaline tide in some dogs.

Compared with the control at the same time points, the potassium citrate supplement increased urinary pH (KCit 1). Significant increases were observed at 7h00, 9h00 and 13h00. A biologically relevant difference was observed at 7h00 and 13h00 (Table 4). During this treatment, two urine samples were lacking for pH analysis due to insufficient volume (day 1‐dog 3–7h00 and day 2‐ dog 5–13h00).

When potassium citrate was supplemented at 10h30 (KCit2) urine pH was significantly lower compared with the control diet at 13h00 but did not differ from supplementation at 7h00 (KCit1) (Tables 4 and 5 and Figure 3).

**TABLE 5:** Estimated differences between means and 95% confidence intervals of specific Time of the urinary pH measured in 7 dogs between control and potassium citrate administered at 10h30–KCit2.

		95% Confidence interval	
	Estimate	2.5%	97.5%
Intercept^a^	7.74	7.32	8.17
Control T6 versus T4^b^	−1.49	−1.96	−1.01^*^
Control T8 versus T4^b^	−2.07	−2.59	−1.55^*^
KCit2 versus Control at T4^c^	−0.23	−0.74	0.28
KCit2 versus Control at T6^c^	0.87	0.35	1.38^*^
KCit2 versus Control at T8^c^	0.45	−0.05	0.96

^a^ Estimated mean in the reference: Control and Time 4 (11h00). T4 is 4 h after the meal (11h00), T6 is 6 h after the meal (13h00), and T8 is 8 h after the meal (15h00).

^b^ Estimated difference between mean of specific Time with reference.

^c^ Estimated difference between mean of specific time for specific treatment with mean of same time of Control.

* Significant and biologically relevant (i.e., pH change of at least 0.5).

KCit2–Potassium citrate administered at 10h30 and 15h00.

Compared with the control at the same time points, the u/d diet increased urinary pH. Significant increases were observed at 9h00, 11h00, 13h00 and 15h00, and were biologically relevant at 9h00, 13h00 and 15h00 (Table 4).

Urical had no effect on urine pH compared to the control (Table 4). During this treatment, one urine sample was lacking for pH analysis due to insufficient volume (day 2‐dog: 5–7h00).

Compared with the control at the same time points, the S/O diet decreased urinary pH. Significant decreases were observed at 7h00, 9h00, 11h00 and 13h00, and were biologically relevant at 9h00 and 11h00 (Table 4).

Table 4 represents the changes in urinary pH compared to the intercept. The absolute mean urinary pH with 95% confidence intervals at the different time points are presented in Supporting Information Appendix Table S1 for the alkalizing supplement (KCit1) and diet (u/d diet), and in Supporting Information Appendix Table S2 for the acidifying supplement (Urical) and diet (S/O diet).

During the trials, we did not observe any changes in faecal scores, despite having no dietary transition periods in between the different diets and supplements.

## 4 DISCUSSION

The purpose of this study was to assess the effect of the oral supplementation of potassium citrate and an ammonium chloride solution (Urical), along with two therapeutic diets, Hill's ® Prescription Diet® u/d® Canine (u/d diet) and Royal Canin® Urinary S/O dog (S/O diet), on a dog's urinary pH, and to evaluate the postprandial alkaline tide effect in dogs. Even though Urical did not produce a significant urinary pH difference compared to the control, results indicated that the oral supplement potassium citrate and the therapeutic dry foods, u/d diet and S/O diet, can influence urinary pH.

Diurnal variation in urinary pH was reported in humans (Cameron et al., 2012). The following studies also demonstrated this event in dogs (Stevenson et al., 2000; Stevenson & Markwell, 2001), although this finding was not always observed (Gleaton et al., 2001). The postprandial alkaline tide results from the secretion of gastric acid in response to food ingestion (Brooks, 1985). As a result of the acid ‘loss’, the kidneys compensate by conserving acid, which consequently produces alkaline urine (Finke & Litzenberger, 1992). Our results show an increase in urinary pH between 9h00 and 13h00 (approximately 2–6 h after food intake) in the control and in all treatments, which confirms the existence of a postprandial tide, whereas urinary pH returned to baseline 8 h after meal (15h00).

Potassium citrate is used for urinary alkalization and the treatment of chronic metabolic acidosis and has a quick and temporary effect on systemic acid‐base status (Papich, 2016b). Results of several human medicine studies show that dietary potassium citrate supplementation given orally significantly increases urinary pH (Doizi et al., 2018; Pak et al., 1986; Preminger et al., 1985). In dogs, a dosage of 40–60 mg/kg BW every 8–12 h is recommended for an alkalinizing effect (Adams & Syme, 2010). Results of another study on healthy dogs indicate that administration of 150 mg potassium citrate/kg BW/day increased mean urinary pH by 0.2 pH units (Stevenson et al., 2000). Nonetheless, this increase was not statistically significant. In our study, the range of dosages of 130–211 mg/kg BW/day dived over two doses per day (2–4 capsules per day to dogs with different BW) and increased significantly the urinary pH by at least 0.5 pH units 6 h after meal (13h00). The observed significant difference in urinary pH from control at 7h00 (time of meal and supplement administration) seems to be a coincidental finding, as it was expected to be similar after an overnight fast. This might be a result of the small sample size and relatively small number of observations. A capsule form of potassium citrate was selected to ensure proper intake in kennel conditions. Nevertheless, it is important to notice the potassium citrate dose variability (+60%) between doses due to this selected form.

The potassium citrate administration at 10h30 (KCit2) aimed to assess the effect of the potassium citrate supplementation apart from the food intake and to evaluate its capacity to prolong the alkaline‐tide effect when administered at the peak of the tide. This effect would result from an increase in urinary excretion of citrate generated by an increment in citrate production inside the mitochondria of renal cells or by a reduction in citrate tubular reabsorption in the proximal tubular cells, as suggested by Stevenson et al. (2000). It was then expected that after the administration of potassium citrate 3.5 h apart from the meal (10h30) the urinary pH would remain elevated for a longer period since the kidney counteracts the resulting metabolic alkalosis with increased excretion of alkaline hydrogen‐carbonate ions (HCO3‐) through the urine (Brooks, 1985; Niv & Fraser, 2002; Rune, 1965, 1966; ). However, this was not observed since there was no biologically relevant difference in urinary pH compared to when given at 07h00. In a study performed on humans with nephrolithiasis, the individuals were kept on a constant diet. The effects of meals on potassium citrate's (2160 mg, three times per day) physiological and physicochemical actions were studied. The result was a significant increase (*p* < 0.05) in the urinary pH, whether given with food or on an empty stomach, with no significant difference between these measurements. Additionally, in the current study, no gastrointestinal side effects were observed with the potassium citrate supplementation.

The u/d diet was selected based on its formulation to offset metabolic acidosis and produce alkaluria with a target pH between 7.1 and 7.7 when fed to dogs. This effect is particularly due to its potassium citrate and calcium carbonate content, as well as the lower protein content of the diet. A mean urine pH of 7.48 after 42 days of treatment was observed by Lulich et al. (2005) when six adult female beagle dogs were fed a canned diet designed to decrease CaOx urolith recurrence (u/d diet), with a 24‐h urine sample collected. In our study, mean urinary pH oscillated between 5.92 and 8.16 when the dogs were fed the u/d diet. It needs to be considered that urinary pH was evaluated over 8 h with frequent pH measurements throughout the day. The methodology used to calculate urinary pH may not be similar, as a target urinary pH for a diet may be obtained in a 24‐h urine pool. In the present study, the group receiving the u/d diet had a higher mean urinary pH for a longer period compared to the group supplemented with potassium citrate. The fact that u/d diet induces a more durable postprandial alkaline tide is unlikely to be due to a higher intake of potassium citrate when the diet is fed, it is more likely to assume that this results from the presence of the extra alkalizing agent (calcium carbonate) present in the u/d diet and eventually to a greater urine acidifying potential of the control diet when compared to the u/d diet. This assumption is supported by Kienzle and Wilms‐Eilers (1994) observations of a longer postprandial urinary alkaline persistence with a calcium carbonate diet (calcium carbonate added to a basal diet of minced beef meat and cooked rice) when compared to the same basal diet without calcium carbonate, and hypothesized it was due to a rather slow absorption (mainly in the large bowel) of the calcium carbonate due to its low solubility. The fact that u/d diet induced a more durable postprandial alkaline can also be due to the different diet composition (e.g., water and/or fibre content) that can affect, for instance, the gastrointestinal transit time.

A few studies claim that ammonium chloride effectively acidifies the urine of dogs when orally administered at 200 mg/kg BW/day (Börkü et al., 1996; Senior et al., 1984; Shaw, 1989). It is recommended to take a dosage of 100 mg/kg BW every 12 h (Papich, 2016a). In the present study, a dosage of 0.5 mL/kg BW/day of Urical (amount indicated by the manufacturer) was used. This dosage corresponds with 9 mg/kg BW/day, which was not effective in lowering the urinary pH of dogs.

In dogs, the S/O diet is marketed for the dissolution of struvite uroliths, to aid in the prevention of struvite and oxalate urolith formation, and to reach a target urinary pH between 6 and 6.5. This diet contains two urinary acidifying substances: calcium sulphate and DL‐methionine. All these claims were considered in the selection of this diet. A couple of studies evaluating the effect of calcium sulphate supplementation in dogs (3.17 g S/kg of diet, Janczikowski et al., 2008) and cats (2.56 g S/kg of diet, Halfen et al., 2018) showed only a small and not significant urinary pH acidification effect, this result might have been due to a low intake of calcium sulphate, which was probably insufficient to produce significant urinary acidification. Multiple studies verified the effectiveness of l‐methionine/dl‐methionine as a urinary acidifier in humans, cats and dogs (Funaba et al., 2001; Halfen et al., 2018; Hickey et al., 2015; Jacobs et al., 2001; Siener et al., 2016;). In the present study, urinary pH oscillated between 5.30 and 6.16 when the dogs were fed the Urinary S/O diet, and although the present study was not designed to determine the impact of any specific nutrient on urinary pH, it is expected that the methionine content of this diet had a major influence on its acidifying effect.

The present study was designed to evaluate the diet impact as a whole and not the effect of any specific dietary nutrient or ingredient on urinary pH. Thus, it is not possible to identify individual contributions, although the results obtained with the diets were most probably influenced by several nutritional components and internal metabolic factors (e.g., hepatic metabolisation and gastrointestinal absorption) and their potential interactions.

The results from the present study were not in total agreement with the diet's target urinary pH, but as discussed previously, the urine samples used to determine urine pH (2‐h samples over 8 h vs. 24‐h pooled sample) are not equivalent.

The small number of dogs can be considered a limitation of this study; however, it was enough to detect significant differences between treatments. Results that might get significant with a larger sample size would be the observed increase of urinary pH on potassium citrate at 9h00, 11h00 and 15h00, and on u/d diet at 11h00, since they were not statistically significant or were not considered biologically relevant. Another limitation is the use of intact male healthy research Beagle dogs, which may respond differently when compared to neutered male dogs, female dogs, dogs of other breeds and/or dogs with a history of urolith formation. Crystal prevention demands urinary pH control over 24 h. Unfortunately, for logistical reasons, it was impossible to monitor the urinary pH for 24 h. It would also have been interesting to have this 24‐h monitoring to assess the effect of the two treatment administrations per day. Instead, our study only allows the evaluation of the 8‐hour periods after the first administration of the day. This limitation is not that detrimental because a similar behaviour is expected to follow the second administration of the day. The short duration of the control phase for 5 of 7 dogs (Part 2) is justified because of the lack of Day effect in Part 1 (Table 3), resulting in quite similar pH measurements among consecutive days (Figure 2). The significant effects of the diets and supplements on urinary pH were already present on day 1 of treatment, and, although each treatment duration was short, significant differences could be detected (Tables 3 and 4). Nevertheless, the variable duration of control phases (Parts 1 and 2), treatments and washout periods can be considered limitations by adding unnecessary complexity to the study. Another limitation is that other metabolic factors affecting urinary pH were not considered. The short‐term nature of the study means that we cannot rule out a different effect with long‐term therapy, as chronic dietary changes or supplementation might result in microbiome changes or other biological changes that are not apparent in the short term.

In conclusion, the present study confirmed that nutrition influences acid‐base balance in dogs. Our study also showed that therapeutic foods were apparently more effective than the administered doses of supplements at influencing urinary pH for the 8‐h postprandial period. The postprandial alkaline tide in dogs was observed approximately 2–6 h after food intake.

The u/d and S/O therapeutic foods used in this study are recommended to induce an alkaline or acidic urinary pH. Urinary pH manipulation is beneficial in multiple conditions but is particularly important in preventing urolith formation, promoting the dissolution of some uroliths such as struvite and cystine. To formulate recommendations concerning feeding management, it is essential to know the effect of food intake on the 24‐h day urinary pH and further studies are required.

## AUTHOR CONTRIBUTIONS

IAI data curation, ALL and RJC conceptualization, JCMV formal analysis, RJC funding acquisition, IAI and RJC investigation, ALL methodology, IAI project administration, RJC resources, ALL and RJC supervision, IAI and ALL writing‐original draft, JCMV and RJC writing‐review and editing.

## FUNDING STATEMENT

This research received no particular grant from any funding agency in the public, private, or not‐for‐profit sectors.

## ETHICS APPROVAL

The study protocol was reviewed and approved by the Clinical Research Review Committee at Utrecht University, registered under number AVD1080020184748, as required by Dutch legislation.

## ANIMAL WELFARE STATEMENT

The authors confirm that the ethical policies of the journal, as noted on the journal's author guidelines page, have been adhered to and the appropriate ethical review committee approval has been received. The authors confirm that they have followed EU standards for the protection of animals used for scientific purposes.

### PEER REVIEW

The peer review history for this article is available at https://publons.com/publon/10.1002/vms3.1285.

## Supporting information

TABLE A.1 Mean urinary pH between 7h00 and 15h00, of 7 dogs, for the control (control diet), and the two alkalizing treatments; potassium citrate supplement (KCit1) with the control diet, and u/d diet, being given at 07h00 and 15h00, right after urine collection.
TABLE A.2 Mean urinary pH between 7h00 and 15h00, of 7 dogs, for the control (control diet), Urical with the control diet, and S/O diet, being given at 07h00 and 15h00, right after urine collection.

## Data Availability

The data that support the findings of this study are available from the corresponding author upon reasonable request.
