# Supplementary material for: Effects of two commercial diets and two supplements on urinary pH in dogs
Source: Vet Med Sci. 2023 Oct 18;9(6):2566–75. doi: 10.1002/vms3.1285 (PMC10650373; doi:10.1002/vms3.1285)
Supplement: Supplementary file 1 — TABLE A.1 Mean urinary pH between 7h00 and 15h00, of 7 dogs, for the control (control diet), and the two alkalizing treatments; potassium citrate supplement (KCit1) with the control diet, and u/d diet, being given at 07h00 and 15h00, right after urine collection. TABLE A.2 Mean urinary pH between 7h00 and 15h00, of 7 dogs, for the control (control diet), Urical with the control diet, and S/O diet, being given at 07h00 and 15h00, right after urine collection. [file VMS3-9-2566-s001.docx]

**Appendix**

**Table 1:** Mean urinary pH between 7h00 and 15h00, of 7 dogs, for the control (control diet), and the two alkalizing treatments; potassium citrate supplement (KCit1) with the control diet, and u/d diet, being given at 07h00 and 15h00, right after urine collection.

| **Time (h)** | **Control** | **Potassium citrate**  **(**130-211 mg/kg BW/day divided over 2 doses per day) | **U/d diet** |
| --- | --- | --- | --- |
| **7h00** | 5.73 [5.43-6.04] | 6.48 [6.11-6.86] | 5.92 [5.55-6.29] |
| **9h00** | 7.59 [7.18-8.00] | 8.02 [7.66-8.39] | 8.16 [7.79-8.53] |
| **11h00** | 7.76 [7.65-8.18] | 7.95 [7.58-8.31] | 8.14 [7.77-8.51] |
| **13h00** | 6.28 [5.85-6.71] | 6.85 [6.48-7.22] | 7.45 [7.08-7.82] |
| **15h00** | 5.67 [5.22-6.12] | 5.93 [5.56-6.30] | 6.14 [5.77-6.50] |
| Control – control diet; Potassium citrate - potassium citrate supplement; u/d diet - Hill's ® Prescription Diet® u/d® Canine | | | |

**Table 2:** Mean urinary pH between 7h00 and 15h00, of 7 dogs, for the control (control diet), Urical with the control diet, and S/O diet, being given at 07h00 and 15h00, right after urine collection.

| **Time (h)** | **Control** | **Urical**  **(**0.5 mL/kg BW/day) | **S/O diet** |
| --- | --- | --- | --- |
| **7h00** | 5.73 [5.43-6.04] | 5.63 [5.26-6.00] | 5.30 [4.93-5.67] |
| **9h00** | 7.59 [7.18-8.00] | 7.82 [7.45-8.18] | 6.16 [5.79-6.52] |
| **11h00** | 7.76 [7.65-8.18] | 7.49 [7.12-7.86] | 6.04 [5.68-6.41] |
| **13h00** | 6.28 [5.85-6.71] | 6.21 [5.84-6.58] | 5.88 [5.51-6.25] |
| **15h00** | 5.67 [5.22-6.12] | 5.70 [5.33-6.07] | 5.46 [5.09-5.83] |
| Control – control diet; Urical - ammonium chloride solution; S/O diet - Royal Canin® Urinary S/O dog. | | | |
